# Structural Adaptation of Cold-Active RTX Lipase from *Pseudomonas* sp. Strain AMS8 Revealed via Homology and Molecular Dynamics Simulation Approaches

**DOI:** 10.1155/2013/925373

**Published:** 2013-05-07

**Authors:** Mohd. Shukuri Mohamad Ali, Siti Farhanie Mohd Fuzi, Menega Ganasen, Raja Noor Zaliha Raja Abdul Rahman, Mahiran Basri, Abu Bakar Salleh

**Affiliations:** ^1^Enzyme and Microbial Technology Research Center, Faculty of Biotechnology and Biomolecular Sciences, Universiti Putra Malaysia, 43400 Serdang, Selangor, Malaysia; ^2^Department of Biochemistry, Faculty of Biotechnology and Biomolecular Sciences, Universiti Putra Malaysia, 43400 Serdang, Selangor, Malaysia; ^3^Department of Microbiology, Faculty of Biotechnology and Biomolecular Sciences, Universiti Putra Malaysia, 43400 Serdang, Selangor, Malaysia; ^4^Faculty of Science, Universiti Putra Malaysia, 43400 Serdang, Selangor, Malaysia

## Abstract

The psychrophilic enzyme is an interesting subject to study due to its special ability to adapt to extreme temperatures, unlike typical enzymes. Utilizing computer-aided software, the predicted structure and function of the enzyme lipase AMS8 (LipAMS8) (isolated from the psychrophilic *Pseudomonas* sp., obtained from the Antarctic soil) are studied. The enzyme shows significant sequence similarities with lipases from *Pseudomonas* sp. MIS38 and *Serratia marcescens*. These similarities aid in the prediction of the 3D molecular structure of the enzyme. In this study, 12 ns MD simulation is performed at different temperatures for structural flexibility and stability analysis. The results show that the enzyme is most stable at 0°C and 5°C. In terms of stability and flexibility, the catalytic domain (N-terminus) maintained its stability more than the noncatalytic domain (C-terminus), but the non-catalytic domain showed higher flexibility than the catalytic domain. The analysis of the structure and function of LipAMS8 provides new insights into the structural adaptation of this protein at low temperatures. The information obtained could be a useful tool for low temperature industrial applications and molecular engineering purposes, in the near future.

## 1. Introduction

A lipase (also known as triacylglycerol acylhydrolase (E.C 3.1.1.3)) is a serine hydrolase, which acts under aqueous conditions on the carboxyl ester bond of triacylglycerol to produce fatty acids and glycerol [1]. Lipases display common *α*/*β*-hydrolase folds that are also present in other hydrolases [2]. A typical lipase consists of an active site comprised of the catalytic triad of serine, glutamine/aspartate, and histidine [3].

Lipases are widely distributed among living organism, including bacteria, eukarya, and archaea as has been reported by Jaeger et al. [4]. Recently, lipases produced by psychrophilic bacteria have been studied because of their low optimum temperatures and high activities at very low temperatures. This is reportedly due to the inherent greater flexibility compared to mesophilic and thermophilic enzymes. These enzymes are severely impaired by an excess of rigidity. Additionally, peculiar properties of psychrophilic enzymes render them particularly useful as valuable tools for biotechnological purposes and for investigating the possible relationships between stability, flexibility, and specific activity [5, 6].

However, the adaptation of the enzyme at low temperatures is not fully understood because there has been little study on psychrophilic enzymes. Some features discovered by scientists include reduced numbers of salt bridges, slightly lower [Arg/(Arg + Lys)] ratios, reduced numbers of nonpolar residues, and higher numbers of exposed nonpolar residues [7]. In term of stability, psychrophilic enzymes are mostly unstable, as has been proven by various demonstrations, including fluorescence spectroscopy and other techniques. The enzyme tends to unfold at lower temperatures and calorimetric enthalpies [8]. Researchers suggest that one feature of these enzymes is higher numbers of nonpolar residues on their surfaces, which is responsible for the destabilization of the water structure surrounding the enzymes. There are also fewer arginine and proline residues; this may increase the backbone flexibility. Note that research regarding the flexibility of the psychrophilic enzymes (using spectroscopic analysis, dynamic fluorescence quenching, and molecular dynamics simulations) has supported the idea that increased flexibility of psychrophilic enzymes contributed to the evolution of psychrophilic enzymes [9]. 

The importance of understanding the structural adaptations of extremozymes is underscored by their usefulness in various industrial applications. Until now, only a few extremophilic organisms, particularly psychrophiles, have been characterized and used as enzyme sources for industrial processes. Previously, a new strain of psychrophilic bacteria (designated strain AMS8) from Antarctic soil was screened for extracellular lipase activity and further analyzed using a molecular approach. Analysis of 16S rDNA showed that the strain AMS8 was similar to *Pseudomonas *sp. A lipase gene named *LipAMS8* was successfully isolated from strain AMS8 with an open reading frame of 1,431 bp that encoded a polypeptide consisting of 476 amino acids. This crude lipase exhibited maximum activity at 20°C. Additional genetic studies revealed that *LipAMS8* lacked an N-terminal signal peptide and contained a glycine- and aspartate-rich nonapeptide sequence at the C-terminus (experimental data). 

In this study, the structure and function of lipase isolated from *Pseudomonas *sp. strain AMS8 are studied by using the structure predicted by using appropriate software. The structural adaptation of the enzyme at low temperatures is also studied using molecular dynamic simulation (MD simulation). Because it is a newly isolated enzyme, further study is needed to provide further understanding and reveal the potential of this psychrophilic enzyme, which may be used for the industrial, biotechnological, and fundamental purposes.

## 2. Materials and Methods

### 2.1. Software

The modeling and simulation of the enzyme's predicted structure was run on a single PC (Intel (R) Core RM i5 CPU, 650 @ 3.2 GHz Co, 4.0 GB RAM) with the Windows 7 Ultimate operating system. The Yet Another Scientific Artificial Reality Application (YASARA) software [10] program was installed on the PC and was used for the molecular modeling and molecular dynamics (MD) simulation of the LipAMS8 predicted molecular structure.

### 2.2. Sequence Alignment of LipAMS8

The amino acid sequence of the LipAMS8 enzyme obtained from NCBI with Accession Number of ADM87309 consists of 476 amino acid residues; a weight of 50 kDa is used for sequence analysis and modeling. The BLAST [11] program identified the homologous sequence that has high sequence identity with the LipAMS8 enzyme. The sequence with the highest score of sequence identity was chosen based on certain characteristics, including the type of origin and the availability of the solved 3D structures. Subsequently, multiple sequence alignment was carried out using the Biology Workbench [12] open software with the protein sequences of *Serratia marcescens* [13] and *Pseudomonas *sp. MIS38 [14] as both of these templates fulfilled the criteria needed as mentioned above.

### 2.3. Comparative Modeling and Validation

The templates used for the modeling were the crystal structures of the lipases obtained from *Serratia marcescens* [13] and *Pseudomonas *sp. MIS38 [14]. The atomic coordinates for the lipases *Serratia marcescens* (PDB ID: 2qua) and *Pseudomonas *sp. MIS38 (PDB ID: 2z8x) were obtained from the Protein Data Bank. The 3D model was generated using the YASARA [10]. The validation was performed with VERIFY3D [15] (to evaluate the fitness of the protein sequence in its current 3D structure) and Ramachandran plot [16] (to evaluate the geometrical aspects of the structure).

### 2.4. Molecular Dynamics (MD) Simulations at Various Temperatures

An MD simulation provided more information for detailed microscopic modeling on the molecular scale. The method follows the constructive approach by mimicking the behavior of molecules with the use of model systems. More powerful computers make it possible to study greater complexity with a realistic expectation of obtaining meaningful and useful information [17]. 

In this study, the MD simulation was performed in water. This involved the simulation of predicted model inside a trajectory box filled with 6940 molecules of solvent (including NaCl and water) and 467 LipAMS8 amino acid residues at the temperatures of 0°C, 5°C, 25°C, 37°C, 50°C, and 100°C. The density of water varies with temperature because the theoretical density of water depends on temperature.

The AMBER03 [18] force field parameter which implemented in the YASARA software was used for MD simulation. In each simulation, the initial model was minimized in order to reduce the contact area difference (CAD) between the protein model and the solvent molecule. During the minimization, conjugate-descent and steepest-descent algorithms were employed.

### 2.5. Simulation Analysis

The enzyme was studied using 240 saved steps for each simulation, which represents up to 6 nanoseconds of the production period. The analysis provides better understanding of the dynamic properties of the enzyme in water at different temperatures. The root mean square deviation (RMSd) was computed for the protein backbone and residues in order to check the stability of the trajectories. Additionally, the root mean square fluctuation (RMSf) was computed per residue in order to study the flexibility of the trajectories. Further analysis was performed by calculating the radius of gyration (Rgyration) and solvent accessible surface area (SASA) of the enzyme within the 6 nanoseconds (ns) of production time. 

## 3. Results and Discussion

### 3.1. Comparative Modeling of LipAMS8

#### 3.1.1. Modeling of LipAMS8

The sequence alignment searches for suitable templates to construct the 3D structure of the lipase AMS8 (LipAMS8), using comparative modeling. In this study, the crystal structures from *Pseudomonas* sp. MIS38 lipase and *Serratia marcescens* LipA (which score 80% and 69% for sequence identity) were chosen as the templates for modeling because both have highest scores of sequence identity when aligned with the LipAMS8 sequence. Both templates also have solved structures that can be obtained from the RSCB PDB Data Bank, which is important for predicting the 3D structure of the enzyme. 

Two templates were used in the modeling of LipAMS8 in such a way that a model was formed from each template, as well as hybrid model which formed based on the best configuration of protein using the two templates chosen. However, the Z-score of each model is the most crucial parameter as the best Z-scores value obtained from each model obtained will denote for the accuracy and the quality of the model itself. Subsequently, the hybrid structure which is expected to be the best model for LipAMS8 is rejected due to poor Z-scores compared to the one obtained using only *Pseudomonas* sp. MIS38 as template.

The model was validated using the Ramachandran plot (Figure 2). The model had 89.5% of the residues residing in the most favored allowed region. Although the best scores are 90.0% and higher, the score obtained is considered to be acceptable because the model is a prediction model and not a crystal structure (i.e., the crystal structure is a fully solved structure compared to the predicted one). In a previous study, the serine of the catalytic triad, which resides in the negative/disallowed region of the Ramachandran plot, was proposed for the active conformation of the enzyme [19]. However, from this study, the Ser^207^ of the catalytic triad of the LipAMS8 resides in the allowed region of the Ramachandran plot. This suggests that the enzyme is in the nonactive conformation, which is supported by the observable lid structure of the enzyme (closed conformation). Prediction of the active conformation of the enzyme also can be performed by observing the lid structure. 

**Figure 1:**
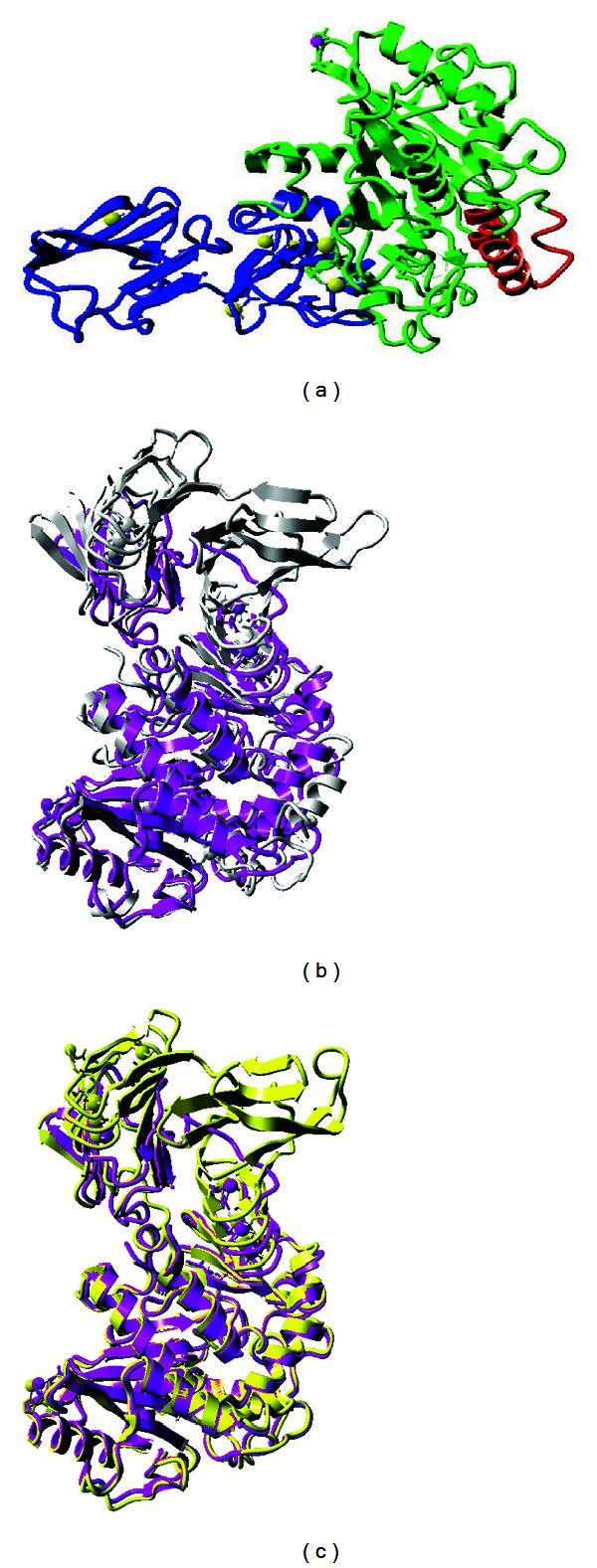
(a) LipAMS8 predicted 3D structure. The structure is composed of catalytic (green) and noncatalytic (blue) domains. The lid is colored in (red). (b) and (c) are superimposition of LipAMS8 structure (purple) with 2QUA (silver), and 2Z8X (yellow).

**Figure 2:**
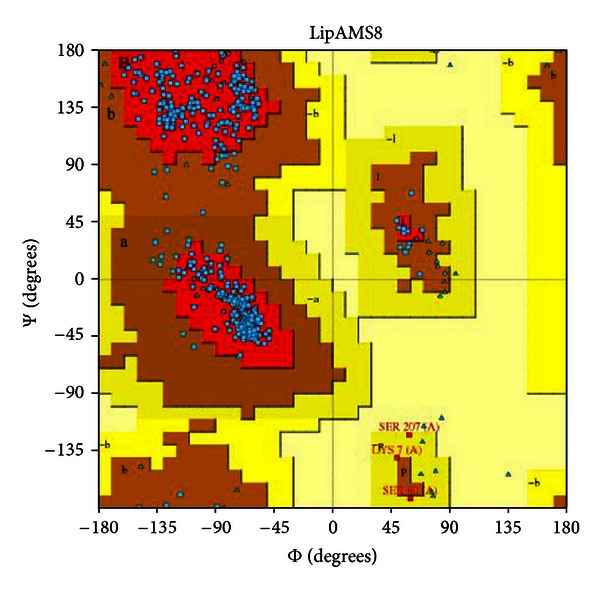
Ramachandran plot of the LipAMS8 3D structure. The structure scores 89.5%, meaning that 89.5% of the residues reside in the most favored region.

As compared to the template structure used to model LipAMS8 as shown in superimposed image in Figures 1(b) and 1(c), the predicted model of LipAMS8 also displays two main regions: the catalytic (green) and noncatalytic (blue) domains, as shown in Figure 1(a). The catalytic domain at the N-terminal is rich in *α* helices, while the noncatalytic domain at the C-terminal is dominated by *β*-strands. The catalytic domain also exhibits the presence of an *α*/*β*-hydrolase fold and catalytic triad, which includes Ser^207^, His^255^, and Asp^313^ residues. The Ser^207^appears in the pentapeptide of G-X-S-X-G motifs (where X represents His^206^ and Leu^208^) and is located at the sharp turn between the *β*-strand and *α*-helix that resembles the nucleophilic elbow (normally present in the structural family of *α*/*β*-hydrolases) [20]. This suggests that the catalytic serine is aided by an oxyanion hole that stabilizes the negative charge generated during a nucleophilic attack by Ser O*γ* [19]. 

Generally, a lipase can exist in 2 conformational states: active and inactive. The lid conformation (open or closed) determines whether the enzyme is in the active or inactive conformation. The active conformation of the lipase is necessary for catalytic activity [19]. In this study, there is a lid-like structure that covers the catalytic site on the catalytic domain, which suggests the inactive conformation of the enzyme. Previous studies suggested that the active form of the enzyme has the lid open, allowing for the entrance of the substrate into the binding site for catalysis [21]. Thus, the closed conformation of the lid in this study meant that the nucleophilic Ser^207^ could not attack the substrate. To open the lid, a water-oil interface is required so the lid can be modulated to uncover the catalytic site, providing access to the catalytic pocket for the substrates [19]. This enhances the activity of LipAMS8. Lid number 1 of LipAMS8 has high numbers of hydrophobic residues, including Ala^51^, Leu^53^, Val^54^, Val^57^, and Val^58^. This may allow efficient interaction between the hydrophobic lid residues with the lipid interface and contribute to easier lid opening.

From this study, the LipAMS8 consists of RTX motifs at the noncatalytic domain, suggesting the LipAMS8 is one of the RTX lipases that belong to the I.3 subfamily. This is further proven by the absence of cysteine residues [22]. Note that, the RTX motif is present in a variety of Gram-negative microorganisms [6]. This motif (comprised of glycine-rich nonapeptide sequences) is usually located at the carboxy-terminal portion of an enzyme [22]. In the 3D structure of LipAMS8, the sequence of RTX motifs constituted the parallel *β*-roll, which the first 6 residues of each motif form to attach calcium ions (Ca^2+^). The remaining 3 residues build short *β*-strands, which result in the right-handed helix of the *β*-strand on the noncatalytic domain. The exact function of the RTX motifs remains obscure. However, they could be receptor binding domains, enhancers of secretion, and internal chaperones [6].

In the predicted model of LipAMS8, there are metal ions, including 6 atoms of Ca^2+^ and 1 atom of Zn^2+^. Note that metal ions are required by a substantial fraction of enzymes to perform catalytic activity. Metal ions may also contribute to substrate activation and electrostatic stabilization of enzyme structure. In this study, Asp^128^, Asp^130^, and the ligand interact in the Zn^2+^ binding sites. The Zn^2+^ present in this LipAMS8 is not located in the catalytic site, so it may not contribute to the catalytic activity of the enzyme. Along with their contribution to catalytic activity, ions may ensure the local and overall structural stability, similar to the function of disulfides [23]. To conclude, there are metal ions in the predicted structure of LipAMS8, which may contribute to the overall structural stability. However, the enzyme is observed to have few or no arginines compared to lysines, low proline content, and a lack of a salt bridge, which reportedly contributes to the adaptation of psychrophilic enzymes at low temperatures. Additionally, the enzyme is predicted to have higher flexibility compared to mesophilic or thermophilic enzymes, allowing psychrophilic enzymes to be active at low energy costs [7]. 

#### 3.1.2. Molecular Dynamic (MD) Simulations

MD simulation was performed to reveal changes in the structure, flexibility, and dynamics of LipAMS8 when simulated in elevated temperatures. The conformational sampling was limited to 12 ns. It has been suggested that LipAMS8 is a cold-active enzyme. Therefore, the structure should denature or unfold as the temperature increases from to 25°C to 100°C because of the disruption of the intermolecular forces, due to the increase in kinetic energy at elevated temperatures. However, in this study, there was no much unfolding of the secondary structure even when the enzyme is simulated at a higher temperature. This might be due to the limitation of conformational sampling, which is a maximum of 12 ns. The simulation time may need to be prolonged in order to see the structural changes.

#### 3.1.3. Molecular Dynamics Simulation Data Analysis

**Figure 3:**
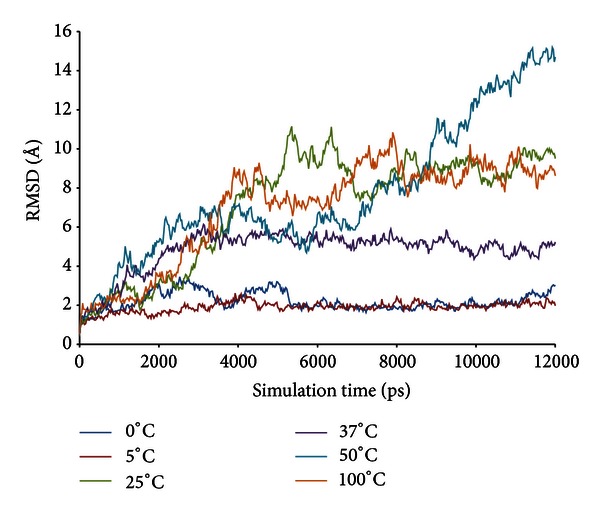
LipAMS8 root mean square deviations (RMSd) of the backbone atoms as functions of time.

The root mean square deviation (RMSd) values of the backbone atoms in the initial models assess the convergence of the protein system. In this study, the RMSd values from the minimized predicted model structure during MD simulation at 0°C, 5°C, 25°C, 37°C, 50°C, and 100°C are shown in Figure 3. At 5°C, the protein remains native-like and equilibrates with an average of 1.83 Å from the minimized structure. This indicates the stability of the enzyme at 5°C. Compared to 5°C, RMSd value at 0°C demonstrated that the 3D structure of the protein becomes unstable when it reaches 1000 ps. The RMSd value begins to fluctuate to values higher than 2 Å and even reached the value of 3.45 Å at 2525 ps. The value dropped to 1.807 Å at 3675 ps and rose to higher than 2 Å at 4000 ps. The unstable fluctuation of the RMSd is consistent with the difference in secondary structure elements observed during the simulation. However, the structural stability is observed after 5500 ps as the pattern of RMSd fluctuation is maintained and does not diverge more than 2 Å towards the end of simulation at 12 ns.

The fluctuation trend for the stability of the enzyme at 25°C, 37°C, 50°C, and 100°C increased in value throughout simulation with RMSd values remaining higher than 4.0 Å at 3000 ps for the rest of the simulation period. The unstable state of this enzyme is supported by experimental data, which found that the enzyme activity of LipAMS8 was reduced when the temperature was increased above 25°C. From the study, we deduce that at 25°C, 37°C, 50°C, and 100°C, the global 3D structure of the protein loses its native structure in order to adapt the molecule to changes in temperature and water density (the solvent). Thus, this result indicates that changes in the geometry coordinates and unfolding of the protein result from the reduced stability of the system. 

We deduced that the most stable temperatures for LipAMS8 simulated in water are 0°C and 5°C as low temperature promotes less conformational movement maintaining structural integrity and stability. On the other hand, for temperatures between 25°C and 100°C, the enzyme structure is unstable and greatly deviates from its initial structure. The higher deviation of the enzyme structure when simulated in water at 25°C to 100°C may be related to the disruption of the molecular forces, which leads to higher kinetic activities of the molecules. However, the structural changes of the enzyme simulated in water are not that critical when compared to the structural changes that occur when the enzyme is simulated in an organic solvent [24]. 

Interestingly, average RMSd scores for 0°C and 5°C was seen to be stable throughout simulation. However, the stability was only adapted to catalytic domain of enzyme which is believed to be promoted by the presence of metal ions such as Zn^2+^ and Ca^2+^. In other hands, the unstable state of this enzyme which is contributed from high flexibility of the noncatalytic domain at low temperature such as 0°C and 5°C however is somehow predictable since the enzyme has to adjust itself towards the low temperature whereby the kinetic energy is slowly decrease. If the enzyme does not increase their flexibility at this point, the structure may become too rigid and could not compensate for the decrease in catalysis temperature.

**Figure 4:**
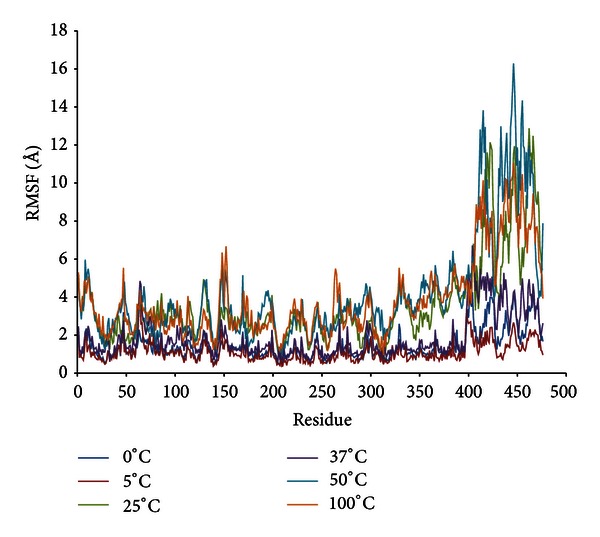
LipAMS8 atoms root mean square fluctuations (RMSf) as functions of time.

Figure 4 shows the root mean square fluctuations (RMSf) per residue for LipAMS8 simulated in water at temperatures from 0°C to 100°C. The average RMSf scores per residue for all temperatures vary from 1.12 Å to 4.1 Å. The highest fluctuation score is at 100°C. This result is equivalent to the effect of higher temperature on the flexibility of the enzyme. However, when comparing the flexibility of the enzyme at 0°C and 5°C, the RMSf value decreased from 1.43 to 1.12 Å. The higher flexibility of the enzyme at the lower temperature may suggest an adaptation of the enzyme to counteract the “freezing effect” as the temperatures dropped.

At the catalytic site, the RMSf scores do not indicate higher flexibility of the residues simulated in water at different temperatures. The flexibility is maintained at a value below the average RMSf score at different temperatures. This suggests the stability of the catalytic triad is supported by the figure of RMSd per residue.

Overall, the pattern of fluctuation per residue was similar to the pattern shown in the figure of RMSd per residue. Both figures show that higher fluctuation occurs at the noncatalytic domain. The most consistent fluctuation across temperatures occurred at residues 393–476, which reside in the noncatalytic domain whereby the RTX repeats are present. The flexibility of the domain may occur because of the presence of high glycine residues, which are known to introduce flexibility in protein structure because they lack side chains [25]. The increased flexibility of LipAMS8 (which originated from the low stability of the noncatalytic domain) may imply that the noncatalytic domain is the crucial part of the LipAMS8 molecular structure. This domain may contribute to high enzyme activity at low temperature. 

An examination of the structural flexibility of the lid structure reveals that lid number 1, comprised of residues 51–58, does not have a higher than average RMSf score at any temperature. However, lid number 2, which comprised of residues 148–167, does have fluctuation at residues 148–153 at most temperatures. This proves the flexibility of the lid residue when simulated in water at various temperatures. This result led to the hypothesis that lid number 2 of LipAMS8 may be able to undergo a conformational transition under the right conditions, such as the presence of substrates. The residues 148–153 on lid number 2, which is more flexible, may acts like a holder that opens up the lid structure to expose the binding site in interfacial activation. In contrast to lid number 1, lid number 2 is more flexible. Thus, lid number 2 may be the first lid to open when substrates are present. The water-oil interface around the opening of first lid is less flexible for binding the substrate to the catalytic site. Note that the lid opening is a crucial step in a lipase with lid-like structure. The flexibility of the residues that reside in the lid structure is important for determining the motion rate of the lid, which plays an important role in the adaptation of enzyme function at low temperatures [7].

The structural flexibility observed on the structure of LipAMS8, simulated in water at various temperatures, may lessen if simulated in organic solvent. A previous study suggests higher rigidity of the enzyme in organic solution [26]. However, there is no much information available at the molecular level to accept or reject the proposal. To check the structural flexibility of the enzyme in both aqueous and nonaqueous environments, further simulation is needed to compare the structural basis of the enzyme.

**Figure 5:**
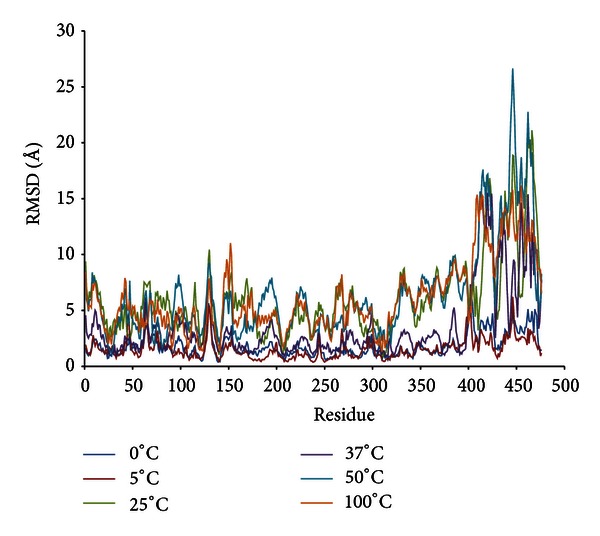
LipAMS8 atoms root mean square deviations (RMSd) per residue.

The root mean square fluctuation (RMSf) analysis per residue and root mean square deviation per residue (RMSd) of the backbone atoms are used to analyze the atomic fluctuations of the predicted model of LipAMS8.  Figure 5 shows the RMSd per residue at 0°C, 5°C, 25°C, 37°C, 50°C, and 100°C. The average RMSd for all residues, including the terminal residue, qualitatively measures the protein flexibility for the relationship between protein conformational flexibility and dynamics.

From the data, the RMSd average values of residues are almost the same between 0°C and 5°C. However, at 25°C, 37°C, 50°C, and 100°C, the RMSd average values increase to 6.29 Å. The pattern of the RMSd average values of residues is similar to the average values of root mean square fluctuation (RMSf) at all temperatures.

The deviation of the structural geometry of the enzyme mainly occurred at the noncatalytic domain. The RMSd values per residue at residues 412–425 and 436–470 are higher than the average score of RMSd at each temperature. From this, the destabilization of the enzyme does not involve the whole protein as proposed by one study [19]. Instead, the destabilization of the enzyme may involve a portion of the enzyme (as observed in this study). Additionally, the data also rejected the postulate that the destabilization of the enzyme mostly occurs at the catalytic domain [8]. The destabilization of the enzyme mostly occurs on the noncatalytic domain; this may be caused by the presence of residues that cause a loss in stability of the enzyme 3D structure. Interestingly, *β*-roll is proposed to maintain the stability of *Pseudomonas *sp. MIS38. Deletion, alteration, and mutation on this *β*-roll which is formed by the presence of RTX motifs and Ca^2+^ would cause the structural conformation changes and denaturation of the enzyme. However, we proposed that actually shorter/lesser number of RTX repeats, less number of metal ions in this counterpart of *Pseudomonas *sp. MIS38, may be the reason why LipAMS8 is able to adapt in extremely cold environment.

Study of the catalytic domain shows that Asp^128^ and Asp^130^, which interact with the Zn^2+^, have RMSd per residue values higher than the average scores of RMSd at each temperature. Thus, Asp^128^ and Asp^130^ are among those residues that fluctuate and mobilize the adaptation to temperature. However, the ratios of fluctuation of Asp^128^ and Asp^130^ to the average scores of the RMSd at each temperature decrease as the temperature increases. This implies higher interaction between metals and Asp^128^ and Asp^130^ at higher temperatures. This result strongly agrees with a previous study, which suggested that Zn^2+^ promotes structural stabilization in an active conformation of the enzyme at elevated temperatures [27]. This result suggests a higher probability of adaptation of the enzyme at higher temperatures, especially in the catalytic domain because Zn^2+^ stabilizes the enzyme structure.

As depicted from Figure 4, the scores for those residues that interact with Ca^2+^ in the catalytic domain are maintained. This indicated that the metal contributes to stabilizing the catalytic domain so that the structure does not deviate from its initial structure. This result agrees with the function of Ca^2+^ in the structural stability of the lipase proposed in a previous study of the *B. glumae* lipase [28]. Experimental data, on the effect of calcium on LipAMS8 activity, also supported the idea that Ca^2+^ may promote structural stability of the enzyme. Thus, the enzymatic properties are maintained and the catalytic activity is improved to provide a greater yield. In contrast to the residues that interact with Ca^2+^at the catalytic domain, Ca^2+^ in the noncatalytic domain has higher values of RMSd and RMSf, which indicates destabilization and flexibility of the residue. The Ca^2+^contribution on the stability of the residue does not agree with the suggestion that it promotes stability. The scores of RMSd and RMSf of the residue that interacts with Ca^2+^ in the noncatalytic domain may be due to the process of adaptation from Ca^2+^ so that the catalytic domain remains stable. This prevents the catalytic domain, which is responsible for catalytic activity, from being unfolded and dysfunction due to changes in the structural stability and configuration.

**Figure 6:**
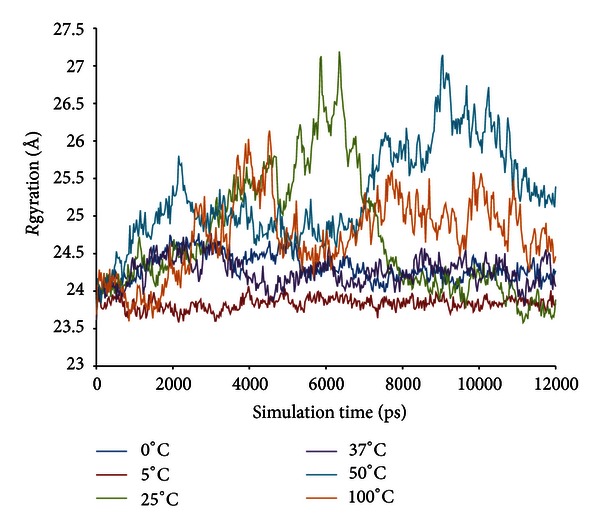
LipAMS8 radius of gyration (Rgyration) scores as functions of time.

**Figure 7:**
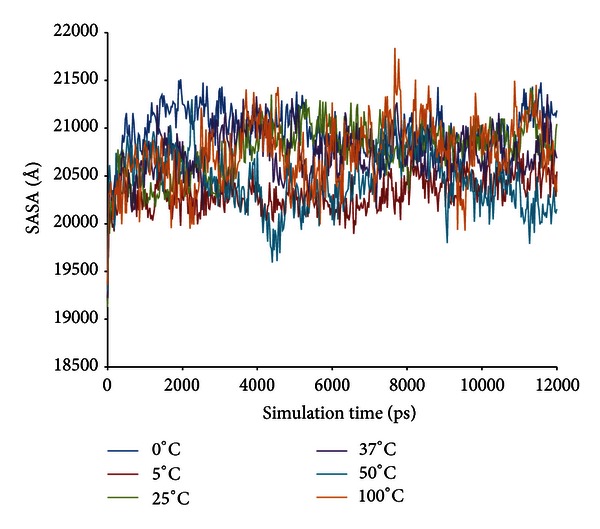
LipAMS8 solvent accessible surface area (SASA) scores as functions of time.

The radius of gyration (Rgyration) of the enzyme at different temperatures within the trajectories at 12 ns is shown in Figure 6. The parameter provides information on the tendency of the protein structures to expand during a dynamic simulation. At 5°C, the score of Rgyration is maintained throughout the simulation compared to the Rgyration score of the enzyme when simulated at higher and lower temperatures. The highest score of radius of gyration of LipAMS8 is at 25°C; the score increased to 27.128 Å within the 12 ns of simulation. This is followed by other temperatures' scores except for the score at 5°C. This indicates the adaptation of the enzyme structure to prevent loss of the native compactness of the structure at low temperature. In general, the result is proportional to the deviation of the enzyme structure, as indicated in Figure 3. While SASA indicates the transfer of free energy required to move a protein from aqueous to nonpolar solvent [29], as exhibited in Figure 7, the SASA scores at 5°C and 0°C show the fluctuation pattern, which is maintained throughout the 12 ns of simulation. Compared to 25°C, the figure increases, which indicates unfolding of enzyme within the simulation period. The unfolding of enzyme at this temperature is proportional to the structural changes of enzyme's secondary structure. The increase in SASA score is also observed at the temperatures of 37°C, 50°C, and 100°C.

From further analysis done, it is observed that the scores obtained from the Ramachandran plot may be able to provide insight about the relationship between enzyme activity and the stability. It is suggested by previous studies from researchers worldwide that 20°C to 30°C is the range for the optimum temperature of cold-active enzyme. Suggesting that, in order for the enzyme to perform the catalytic activity optimally at particular temperature, the enzyme should be in good conformation whereby the degree of quality of the enzyme may be judged based on the scores obtained from the Ramachandran plot. As stated in Table 1, structure obtained from 25°C scores for the highest percentage of Ramachandran plot compared to others indicates the best geometrical conformation of the enzyme at this temperature. On the contrary the stability judged from the figure indicates the instability of enzyme at 25°C. The contrast result from Ramachandran plot and average RMSD scores is found to be the key to explain how the structural stability and conformation could give impact to the catalytic activity of the enzyme.

Noting that, in cold-active enzyme the issue on the enzyme dynamic stability and activity remained as hot issue to be discussed among cold-active enzyme researchers. As observed, at 25°C the enzyme is in a good geometrical conformation which may allow for the best activity at this temperature even though the enzyme is seen to be unstable at this temperature. From these, we deduce that flexibility of the enzyme is crucial to allow the catalytic activity of the enzyme. In contrary to mesophilic and thermophilic counterparts, if the cold-active enzyme is too rigid or too stable with low flexibility, the low activation free energy value (Δ*G* ^#^) may not be able to be overcome so that the activity cannot be promoted and thus leads to inability of the enzyme to perform the catalytic activity.

As observed in all lipases, the common *α*/*β*-hydrolase fold which is normally seen in lipases structure is also observable in the structure of both LipAMS8 and T1 lipases [30]. Both have lid structures that proposed to function like doors that will open up during interfacial activation. Noting that both LipAMS8 and T1 differ in terms of their philicity towards temperature, significance difference in stability towards certain temperature is observed. LipAMS8, due to its psychrophilic properties, is suggested to be stable at 0°C and 5°C with optimum temperature for activity around 25°C as annotated by the pattern of average C*α* atom root mean square deviation throughout 12 ns simulation period. Compared to T1 lipase which is stable at 60°C with optimum temperature of 70°C [31], LipAMS8 has the ability to function at low temperature whereby no other thermophilic enzyme is able to function. The mechanism of adaptation for LipAMS8 enables it to withstand and maintain the stability of the enzyme even at low temperature. However, to concise that eventhough LipAMS8 is able to maintain the stability at low temperature, LipAMS8 also is prone to cold denaturation due to broken bond and interaction of molecules. In other hands, T1 lipase which is thermoalkalophilic enzyme is more stable at higher temperature due to the presence of numbers of metal ions such as Zn^2+^ and Ca^2+^ that are proposed to promote stability towards the T1 lipase.

## 4. Conclusions 

The structural and dynamic study of LipAMS8 provides insights on the structural properties of the enzyme when simulated in water at various temperatures. This RTX (repeat in toxin) lipase might be the first psychrophilic lipase studied in terms of structural stability and flexibility using MD simulation. The enzyme is in a closed conformation and inactive as indicated by the presence of closed lids. From the MD simulation, the LipAMS8 has higher stability at 0°C and 5°C; the catalytic domain exhibits higher stability but lower flexibility compared to the noncatalytic domain. The structural change mainly occurs on the noncatalytic domain of the enzyme, including the unfolding of the secondary structures. 

There has not been much work on the physicochemical properties of the enzyme. Therefore, further study, including crystallization of the enzyme, simulation of the enzyme in organic solvent, and docking, should be performed to provide information and understanding of the cold adaptation and structural insights of LipAMS8.

## Figures and Tables

**Table 1:** Ramachandran plot scores for LipAMS8 after simulation for 12 ns at different temperatures.

Temperature	0°C	5°C	25°C	37°C	50°C	100°C
Most favored regions (%)	85.1	83.1	87.2	81.3	84.4	82.1
Additional allowed regions (%)	13.1	15.9	12.3	17.7	14.9	16.2
Generously allowed regions (%)	1.5	0.8	0.3	0.3	0.0	1.3
Disallowed regions (%)	0.3	0.3	0.3	0.8	0.8	0.5
Nonglycine and nonproline residues (%)	100.0	100.0	100.0	100.0	100.0	100.0
Endresidues (excl. Gly and Pro)	2	2	2	2	2	2
Glycine residues	69	69	69	69	69	69
Proline residues	15	15	15	15	15	15
Total number of residues	476	476	476	476	476	476

## Abbreviations

Å: Angstrom
ns: Nanoseconds
ps: Picoseconds
LipAMS8: Lipase AMS8
Rgyration: Radius of gyration
RMSd: Root mean square deviation
RMSf: Root mean square fluctuation
RTX: Repeat in toxin
SASA: Solvent accessible surface area
YASARA: Yet Another Scientific Artificial Reality Application.
